# “Esa extraña marca de los más célebres bandidos”: el tatuaje en Colombia en la primera mitad del siglo XX

**DOI:** 10.1590/S0104-59702025000100009

**Published:** 2025-05-02

**Authors:** Miguel Adolfo Galindo Pérez

**Affiliations:** i Doctorando, Lateinamerika-Institut/Freie Universität Berlin. Berlín – Alemania. miguel.galindop@outlook.com

**Keywords:** Tatuaje, Crimen, Antropometría, Prisiones, Colombia, Tattoo, Crime, Anthropometry, Prisons, Colombia

## Abstract

Este artículo realiza una aproximación a la historia del tatuaje en Colombia en la primera mitad del siglo XX. Se analiza la racionalización de una serie de estigmas policiales y médico-legales en debates locales que caracterizaron al tatuaje como un elemento de diagnóstico del crimen y la enfermedad. Estas lecturas permitirán observar aspectos generales del proceso de apropiación del tatuaje como práctica artística incluyendo sus contextos sociales y visuales de producción, las características de tatuadores y tatuados y las técnicas de tatuado ejecutadas. Así, podrá evidenciarse al tatuaje dualmente como un insumo de las estrategias de vigilancia y como una expresión de los grupos subalternos y criminalizados de principios de siglo.

## Abstract (en)

This article focuses the history of tattoos in Colombia in the first half of the twentieth century, analyzing the rationalization of several police and medical-legal stigmas in local debates that characterized tattoos as a diagnostic element of crime and disease. With these readings, we will observe general aspects of the process of appropriation of tattoos as an artistic practice, including their social and visual contexts of production, the characteristics of tattoo artists and tattooed people, and the tattooing techniques performed. Thus, tattoos can be seen both as an input for surveillance strategies and as an expression of the subaltern and criminalized groups of the beginning of the century.

## “La banda de apaches”

No hay necesidad de preguntarles de dónde han venido. Por sus tatuajes sabemos que son ustedes evadidos del penal francés.

Charrière (1973, p.569)

En la mañana del martes 8 de enero de 1919, falleció un sujeto no identificado en el Hospital San Juan de Dios de Bogotá tras ser ingresado apenas unos minutos antes por dos agentes de vigilancia que lo habían cargado en hombros desde los calabozos de la Policía Nacional. La noche anterior este individuo desconocido había sido el protagonista de un intento de robo en la casa de la señora Zoila Santos de Caipa, en la calle de la Cochera, en el Centro de la capital. De acuerdo a lo narrado por la prensa bogotana, alrededor de la media noche, el sujeto junto a dos compinches había intentado ingresar por el tejado de la casa de la señora Santos mientras ella dormía. Para su infortunio, los ruidos de los tres ladrones despertaron a una Zoila que, alarmada ante la presencia de extraños en su casa, corrió a la puerta principal gritando y pidiendo ayuda para despertar a los vecinos y llamar la atención de los agentes de policía que patrullaban la zona en la noche (La muerte…, 9 ene. 1919, p.2; La banda de apaches…, 10 ene. 1919, p.3).

Según testigos, al ver llegar a los policías, dos de los sujetos lograron huir, mientras que nuestro protagonista, que había quedado en el tejado, abrió fuego con un revolver para repelerlos y precipitarse en fuga, con la mala suerte de que una teja se atravesó en su camino, haciéndolo resbalar y desplomarse en el pavimento, donde quedó inconsciente. El caído, que presentaba una hemorragia nasal y una contusión en la sien izquierda, considerada no muy grave por los agentes de vigilancia que creían que fingía estar agonizando, fue cargado a los bretes de la central de la policía, donde pasó toda la noche en un estado comatoso. Al amanecer del día siguiente, viendo que su estado no mejoraba, fue trasladado al Hospital San Juan de Dios, donde fue declarado muerto (La muerte…, 9 ene. 1919, p.2; La banda de apaches…, 10 ene. 1919, p.3).

Este caso pudo haber ocupado apenas unas líneas en la prensa local, acostumbrada a tratar a diario todo tipo de crímenes de sangre y contra la propiedad. Pero, más allá del hecho criminoso, el cuerpo inerte de Jorge Cortés, sobrenombre con el que la policía lo identificó, llamó la atención de tal manera que el caso preocupó considerablemente tanto a detectives como a la prensa misma, que le dedicó portadas y páginas enteras durante varias semanas. El sujeto depositado en la morgue del hospital era un extranjero de rasgos europeos, un “hermosísimo mancebo de 30 a 35 años”, al que el soplo de la muerte no le logró alterar su escultura de “joven bandido bello y sereno”. Para los reporteros este era un ejemplar masculino tan perfecto que, a diferencia de los demás muertos que pasaban por la morgue, era incapaz de causar algún tipo de repulsión, pues su apariencia era la de alguien plácidamente dormido, no la de un muerto. Como lo describió el diario *El Tiempo,* este era “esbelto, el tórax ampliamente desarrollado, la cintura delgada, recia la musculatura, el rostro de lineamientos firmes, la boca finamente diseñada, la nariz del más aristocrático corte, la frente digna de una escultura griega” (La muerte…, 9 ene. 1919, p.2).

La fascinación por su belleza y el enigma de la identidad se vio alimentada por un elemento extra en el cadáver, pues unas misteriosas manchas, es decir, unos espectaculares tatuajes lo adornaban por todas partes. Para muchos ciudadanos, e incluso algunos especialistas de las ciencias médicas, jurídicas y policiales, este era un fenómeno desconocido en Colombia, del que apenas habían leído en notas recogidas de prensa extranjera, especialmente aquella que abordaba esta práctica como algo común en presidios y colonias penales de ultramar, así como en manuales medicolegales que teorizaban en torno a la identidad, el tatuaje y su relación con lo biológico y cultural en el individuo.

Como lo apunta Gemma Angel (2013), de no ser por las investigaciones hechas por estos profesionales médicos, judiciales y policiales preocupados por comprender las causas del crimen, la historia del tatuaje en Europa y en el continente americano sería bastante opaca. De esta manera, este artículo tiene como objetivo analizar la historia del tatuaje de principios del siglo XX en Colombia, evocando a sujetos y prácticas inmersas en su producción, aproximándonos a la piel tatuada como un objeto material, susceptible de ser contextualizado dentro de unas formas de producción artística y material, y analizando la racionalización de teorías y estigmas en torno al mismo dentro de las ciencias jurídicas, médicas y policiales.

De esta manera, la investigación está organizada en tres secciones que parten con una aproximación a la historia del tatuaje que evalúa sus orígenes y su proceso de apropiación en Colombia a través de determinados agentes y espacios de realización, discutiendo para ello los cruces entre tradición indígena y flujos migratorios. La segunda parte aborda la elaboración de las primeras discusiones hechas por las ciencias criminales sobre el tatuaje en Colombia a principios del siglo XX, en las que destacan la asignación de valores e imaginarios negativos a las primeras expresiones de esta forma de marcación. Finalmente, en la tercera parte nos ocuparemos del estudio de las dos principales investigaciones hechas sobre el tatuaje en el país que ofrecen valiosas claves de lectura para comprender los objetivos citados en el párrafo anterior.

En la historiografía existe una amplia variedad de estudios que buscan averiguar las relaciones entre marcación corporal, criminalidad e identidad desde la Antigüedad hasta la configuración de los Estados-nación contemporáneos, principalmente en EEUU, Europa y sus colonias (Caplan, 2000a; Putzi, 2006; Cole, 2001; Deter-Wolf et al., 2016; Martel, Larsen, 2022; Anderson, 2004; Rocha, 2022; Guerzoni, 2018). Estos han problematizado en torno a las ideas centrales de este texto, a saber, la elaboración de teorías salubristas y criminológicas en torno al tatuaje, y, atendiendo al perfil de los individuos tatuados, indagan en sus genealogías los espacios y formas de producción, y los circuitos de difusión del mismo.

En América Latina la historia del tatuaje ha llamado la atención de varios académicos en los últimos años que han analizado las temporalidades y expresiones del tatuaje puestas en relación con las conductas criminales. En su mayoría se ha tratado de trabajos enfocados en el estudio de las ciencias medicolegales, policiales y de prisiones, en las que, si bien el tatuaje no ha sido protagonista, si se ha evidenciado como un elemento coyuntural en la construcción de estos saberes y prácticas (Galeano, 2018; Galeano, Schetinni, 2019; García Ferrari, 2010; Palacios, García Ferrari, 2017). Situación replicada en el caso colombiano, donde es referenciado para explicar procedimientos policiales, corrientes criminológicas, historias de presidios y en algunos estudios sociológicos y antropológicos sobre el mismo en la actualidad (Romero, 2016; Rueda, 2015; Hering, 2019; Ortiz, 2012; Cardoso, 2006; Alzate, 2012; Gómez, 2012; Galindo, 2025; Pérez, 2009).

Junto a estas, destacan otras investigaciones que, para el caso brasileño y mexicano, han ocupado al tatuaje como objeto de estudio para recrear, por una parte, los usos populares del mismo a partir del siglo XVI, especialmente en la región de Nueva España. Estas han evidenciado al mismo como un elemento originario dentro de los pueblos indígenas del continente que entró en contacto con las culturas del tatuaje europeas, asiáticas y oceánicas tras el contacto con la colonia. Así, la presencia del mismo ha sido vista como parte de tradiciones devocionales religiosas integrantes de la cultura popular, y como un elemento implementado dentro de prácticas de control y vigilancia apropiadas para definir las maneras de observar el cuerpo tatuado a finales del siglo XIX (Moreno Silva, 2011; Rodríguez Luévano, 2016; Jeha, 2019).

Las claves de lectura ofrecidas desde estas aproximaciones serán útiles para discutir los objetivos propuestos en esta investigación con los cuales se quiere aportar a la literatura existente haciendo un análisis de la historia del tatuaje como forma de marcación irreversible, como se conoce hoy día. Para ello se revisarán las formas y circuitos de apropiación del tatuaje, de sus prácticas y tecnologías, y se identificarán los espacios de realización a partir de estudio de textos que en su momento se preocuparon por realizar interpretaciones científicas en torno a la corporalidad del individuo tatuado. Para ello, se realizará un diálogo entre un limitado pero valioso acervo archivístico en su mayoría, a mi saber, inédito, compuesto por informes de prensa, fuentes policiales (revistas, fichas policiales, fotografías de reos etc.), discusiones medicolegales, dibujos de tatuajes y recortes de piel tatuada conservados en museos. Estos últimos, sobre los que se hará una primera aproximación.

No obstante las novedades documentales acá presentadas, se hace necesario reconocer la ausencia de documentación que referencien las posturas provenientes de los sujetos tatuados. Al ser estos generalmente integrantes de las clases sociales menos favorecidas, sus vidas escapan de la documentación convencional. Por lo que, aprovechando algunas breves claves sobre sus trayectorias, actividades económicas y vinculaciones personales tomadas de la prensa, se quiere dar cuenta de algunos aspectos silenciados en los registros oficiales al tatuaje, su elaboración, sus motivos, su aprendizaje etc. en un periodo de carácter indicativo. Esto, en la medida que no se pretende hablar de la historia del tatuaje con un inicio y un final, ya que, como apuntó Dominique Kalifa (2018) a propósito de los bajos fondos, no existen fechas para delimitarlos con la precisión de los regímenes políticos y económicos. Sino como una forma de dar cuenta de los usos, prácticas y espacios del tatuaje dentro de un determinado periodo de reflexiones colectivas y científicas que, si bien han tenido un inicio y un final en el tiempo, como veremos al final, no desaparecen totalmente. En tanto que sus postulados, desanudados hacia finales de este periodo, quedaron disponibles y son reutilizados en reconfiguraciones sociales y políticas, y por las mutaciones de las representaciones frente al crimen y la exclusión.

## [Id]entidades del tatuaje

Su nombre, evidentemente, no era Jorge Cortés. Poco después se supo que era un francés que de día se ganaba la vida vendiendo dulces en una mesa en San Victorino. En las pesquisas hechas en su habitación, la policía halló varios objetos de valor robados al mismo propietario del arma que accionó antes de caer, así como capuchas, llaves inglesas y lo que al parecer era una llave maestra para abrir cerraduras. En el bolsillo de su pantalón, en una carta indescifrable escrita en *patois*, la policía identificó palabras en francés como *mort*, *hier*, *tête de chien* y *diable*, que fue reconocido por los detectives como un argot de presidio. Por lo cual se planteó que, junto a los otros dos que lograron huir, fuera un fugado de las colonias penitenciarias de la Guayana Francesa (La muerte…, 9 ene. 1919, p.2; La banda de apaches…, 10 ene. 1919, p.3). Ante la posible presencia de un temible criminal internacional se procedió a un proceso de identificación del cadáver hecho por el capitán José Osuna Pineda, miembro de la Guardia Civil española y conocedor de los fenómenos criminales europeo, que se encontraba en Colombia desde 1916 instruyendo a la Policía Nacional en técnicas de investigación, identificación criminal y estrategias de vigilancia (Galindo, 2025).

Para registrar e identificar el cadáver, Osuna implementó el sistema de registro antropométrico judicial que por entonces estaba instruyendo en algunas capitales del país. Este sistema, desarrollado por Alphonse Bertillon en la policía parisina hacia finales del siglo XIX, fue un método de identificación de criminales, prófugos y reincidentes, basado en registro corpóreo y fotográfico de relapsos y sospechosos. Esta era una “danza elaborada”, como la llamó Simon A. Cole (2001, p.10-11), que contaba con un lenguaje científico para describir hasta 11 distintivos morfológicos. Este lenguaje se integró como un poder de escritura y observación en los engranajes policiales de los que surgió una serie de códigos de individualidad que permitían homogeneizar y transcribir los rasgos individuales establecidos en el examen físico de la señalización. Este procedimiento escritural permitió construir al delincuente como objeto descriptivo y analizable en rasgos específicos, por lo que el tatuaje representó una solución científica al problema insoluble de la identificación criminal, siendo un elemento inequívoco dentro del proceso antropométrico por su capacidad de delación y por ser inconfundible (Foucault, 2009; Henry, 1913).

Supliendo como pudieron la ausencia de herramientas para el registro morfológico del cadáver, Osuna y el agente Nieto lograron en tres horas realizar la ficha antropométrica del difunto, examinando cada detalle y dibujando a mano los tatuajes hasta dar con una descripción más o menos completa. Entre cicatrices y marcas de sífilis, Osuna detalló los tatuajes que, a su parecer, eran los más hermosos que había visto desde que era Guardia Civil, ya que, por su fineza y perfección, creía que eran realizados en París, “única parte del mundo en donde los tatuajistas han alcanzado a realizar estas maravillas” (La muerte…, 9 ene. 1919, p.2):

En la espalda se encontraba la mejor parte tatuada. Mostraba un irreprochable dibujo hecho con tinta china, que medía 35 centímetros de alto por 36 de ancho. Representan un tapiz con orla en su parte superior y derecha. En este tapiz figura un juicio de Dios, un torneo entre caballeros de la Edad Media. En la parte derecha se ve un caballero con armadura cabalgando en brioso corcel y en la parte izquierda inferior se observa un heraldo. En la superior derecha se ve una especie de palco en las que se perciben catorce figuras de hombres, mujeres, etc., etc. En su parte izquierda inferior hay una muralla almenada, varias torres y un castillo, varios caballos con armaduras y lanzas. En el brazo derecho tiene un tatuaje de 17 centímetros de largo por 9 de ancho, representa un ramo con ocho hojas; más abajo se encuentra otro tatuaje consistente en una línea azul de 14 centímetros de largo por 4 de ancho. En seguida de esta se ve una mujer con falda estrecha, y al lado de este otro tatuaje que representa a un caballero con levita, bastón y guantes (La muerte…, 9 ene. 1919, p.2).

La ficha descriptiva del cadáver fue enviada por canales diplomáticos a Europa con el fin de averiguar los antecedentes del desconocido al que “la muerte selló en sus labios importantísimos secretos” (La muerte…, 9 ene. 1919, p.2), y cuyo cuerpo había logrado en apenas unos días intrigar a cronistas, policías y a todo aquel que lo contempló.

Tan solo unas semanas después la Direction de la Sûreté Générale en París lo identificó. La capacidad enunciativa de su cuerpo lo delató, ya que la búsqueda alfabética de su nombre, burdamente castellanizado por la policía colombiana, no dio resultados. El lenguaje universal de la antropometría se manifestó: los cuerpos femeninos, las cabezas de payaso, las estrellas, los tatuajes borrados a la fuerza etc. indicaron que en realidad se llamaba Georges Petit, un apache famoso en París y en Marsella, que había sido condenado a trabajos forzados a perpetuidad en el presidio de Cayena por robo y reincidencia (Osuna, 1929; Los apaches…, 13 sep. 1919). En una hazaña que antecedió a la de “Papillon” en los años 1930, Petit se fugó de Cayena en 1916 hacia Maracaibo, para luego pasar a San Antonio del Táchira y de allí a Colombia dedicándose, de acuerdo a los informes diplomáticos, “a propagar en provecho propio las ideas anarquistas” en Venezuela y Colombia (Télégramme 44, 10 nov. 1919, p.14; Tanet et Dortet…, 1920).

Los apaches eran, pues, una suerte de subcultura criminal surgida en la Francia de la *Belle Époque*, definida por muchos como un ejército compuesto tanto por simpatizantes anarquistas como por simples criminales tatuados que sembraban el terror en los barrios parisinos. Iban marcados en sus pieles como sus homónimos nativos norteamericanos, que, por su profesionalismo para cometer robos, asesinatos y atentados, fueron comparados por la prensa colombiana con los “violentos araucanos” y con los indios pijaos. Su fama trascendió a Belleville, Montmartre o Charonne llamando la atención de policías internacionales y criminólogos que quisieron infructuosamente prevenir su llegada a Latinoamérica como consecuencia de sus continuas fugas del presidio ultramar de la Guayana Francesa (Galindo, 2025).

La escalofriante noticia que confirmó la presencia de un prófugo de Cayena en la capital aumentó cuando la policía confirmó la captura de cinco ciudadanos franceses en medio de una *razzia* contra todos los extranjeros que levantaran alguna sospecha de ser los cómplices de Petit y que no se ciñeran al modelo de migrante deseado, de raza centroeuropea con una profesión, buenos antecedentes y costumbres, que se anteponía al agitador sospechoso indeseable moral e higiénicamente (Martínez, 2001). Entre estos cinco presuntos prófugos, denominados por la policía como “La banda de apaches”, en referencia a aquella subcultura criminal de individuos tatuados “célebres por su ferocidad” que camparon durante la *Belle Époque* en los barrios parisinos, figuraban un fraile franciscano, un joyero, dos soldados franceses de la Guardia Colonial, un obrero y un marinero (Los apaches…, 13 dic. 1918, p.2; La banda de apaches…, 10 ene. 1919, p.3; Rocha, 2014).

Algunos de estos estaban tatuados con las más excéntricas figuras, flores y bellas mujeres, despertando en muchos observadores admiración por la belleza estética de estas marcas que, como apuntó un reportero de *El Nuevo Tiempo*, era “el tatuaje más perfecto de cuantos hemos contemplado” (La banda de apaches…, 10 ene. 1919, p.3). Para los detectives colombianos la situación era más compleja, pues marcas con frases como *amour*, *pour vie* y *pas de chances* generaron grandes sospechas por sus posibles peligrosas significaciones. Sin embargo, para los tatuados el problema era mucho más simple que las elaboradas teorías planteadas por muchos científicos. Ante la pregunta del jefe de investigación criminal a uno de los presuntos apaches sobre por qué se había tatuado, respondió que fue producto del aburrimiento durante su tiempo libre en la Legión Extranjera en África, y que lo había hecho “*pour passer le temps*” (La banda de apaches…, 10 ene. 1919, p.3).

**Figura 1:**
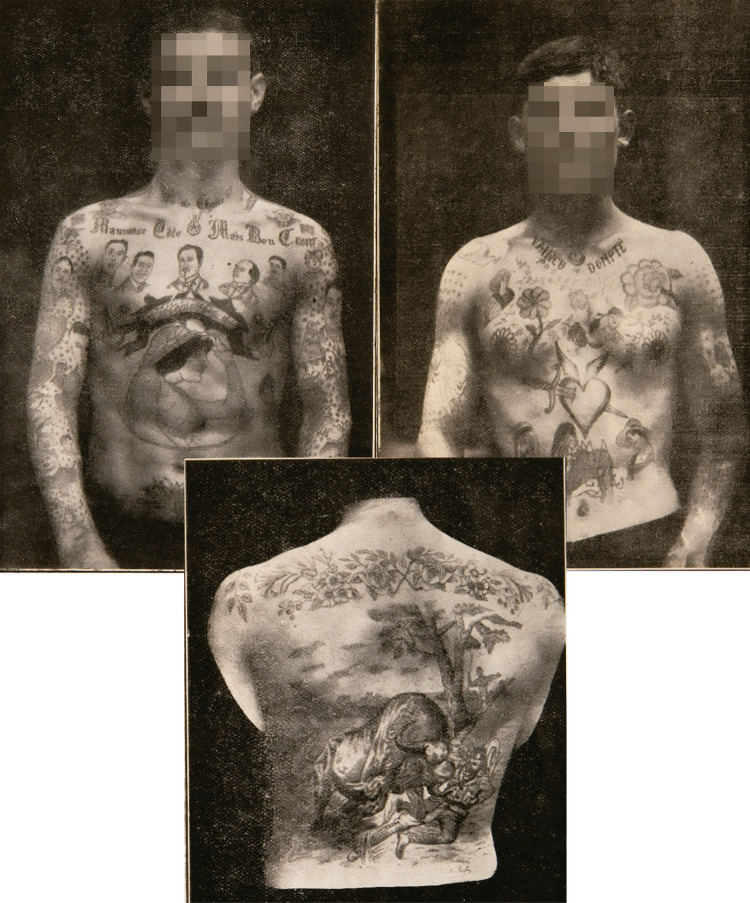
: Tatuajes de “apaches” fotografiados por Osuna (1929, p.298-301)

No contentos con esta explicación, a propósito de este caso se empezaron a plantear diversas discusiones en torno al tatuaje. El diario *El Nuevo Tiempo* señalaba que “esta extraña marca de los más célebres bandidos” no era una costumbre en Colombia. La simple idea de que alguna persona se dejara dibujar cosas terribles en el cuerpo, soportando para ello dolores atroces, solo podía suceder en pueblos salvajes de la Melanesia, en aborígenes australianos, entre habitantes de la Tierra del Fuego, así como en los marineros y criminales “camorristas” italianos (La banda de apaches…, 10 ene. 1919, p.3).

Esta afirmación generalizada entre periodistas, médicos, abogados y público en general no era cierta. Aunque sería problemático pretender periodizar el origen del tatuaje en Colombia, puede decirse que este fue una realidad en la región mucho antes de que el cuerpo desnudo de Georges Petit levantara cualquier interrogante. El tatuaje no “llegó” al continente americano tras la conquista española en los cuerpos de los europeos tatuados o de los esclavos africanos marcados con escarificaciones tradicionales y con quemaduras de fuego hechas por los esclavistas antes de embarcarlos en África. Al igual que otros pueblos de la Antigüedad, los que entonces habitaban la región ya lo practicaban (Jeha, 2019; Rueda, 2015; Deter-Wolf, 2024).

Este fue un saber ampliamente conocido por las comunidades amazónicas Campa, Chayahuita, Amarakaire, Lama-Napo, entre muchos otros, que llegaban a tatuarse en diversas partes del cuerpo (Montoya, 2014; Vivar, 2014). Si bien el tatuaje indígena merece una investigación aparte, debe señalarse que el tatuaje como muestra de identidad entre los pueblos originarios existió también en Colombia por muchos siglos. Y aunque las pinturas faciales sin perforación de la dermis primaron entre los yujos, nukaks, piapocos, tikunas, guahibos y wayuús, estos últimos contaron con sus propias tradiciones de marcado corporal permanente, aunque las memorias de fuga de “Papillon” indicaran que el tatuaje era entonces desconocido entre los guajiros (Naranjo et al., 2012; Charrière, 1973). Por una parte, con el llamado “Asho’ojushi” (“puyado con espina”, en el idioma wayuunaiki), tatuaje hecho con espinas de cactus y carbón vegetal del árbol trupillo mezclado con agua para marcar el símbolo de su clan en brazos piernas y mentones principalmente entre mujeres, encargadas de hacer los tatuajes (Guerra, 2012; Uriana, 31 ago. 2023); y, por otra parte, la marcación de figuras de animales totémicos que definían castas con la aplicación de hierro candente sobre la piel que dejaban una cicatriz queloide (Weston, 1937; Robledo, 1924; Hernández, 1936).

Pero, a pesar de la existencia de estas marcas entre los pueblos nativos, al igual que lo sugerido en el caso mexicano, el tatuaje colombiano de inicios del siglo XX que nos ocupa, además de ser parte de una tradición indígena, su apropiación parece estar relacionada con la movilización de saberes y prácticas extranjeras. Esto pudo deberse a la lejana posición geográfica de estas comunidades ubicadas en una “esquina aislada de Colombia”, de lo que se puede inferir dificultades en la trasmisión de sus tradiciones más allá de sus territorios. Todo esto enfrentado además a las iniciativas evangelizadora realizadas en la región desde la época colonial y hasta el siglo XX que pretendieron civilizar poblaciones y transformar sus modos de vida y tradiciones consideradas “salvajes” (Weston, 1937; Guerra, 2012). En este sentido, como se ha apuntado desde la antropología, pese al conocimiento que se tenía sobre el tatuaje indígena, los puentes entre el tatuaje y el mundo occidental se empezaron a construir solo cuando los marineros, los viajeros, los prófugos y los comerciantes marcaron sus propias pieles (Pérez, 2009).

Esta es una idea generalmente sostenida por los historiadores del tatuaje que indica que la historia del tatuaje occidental está característicamente llena de relaciones entre marineros y criminales. Y que esta ha sido moldeada por encuentros con prácticas de tatuado de todo el mundo como parte de una fase de peregrinación por los sectores marginales de la sociedad (Lodder, 2022). Allí, entre presos, prostitutas y soldados es donde empieza la circulación del tatuaje y, por ende, cuando cobra importancia al convertirse en objeto de preferencia entre estos sectores (Pérez, 2009). Son estos sujetos, apaches como Petit, producto de los bajos fondos europeos y americanos, los agentes responsables de la circulación de este saber. Del que aprendieron sus técnicas sometiéndose a tan doloroso procedimiento y que luego replicaron en sus compañeros de viajes y encierros proliferando este saber a partir del periodo colonial aprovechando la progresiva mejora en los medios de transporte y comulaciones. Lo cual hizo que esta circulación de individuos, materiales y conocimientos convirtiera a regiones como Latinoamérica no en bloques homogéneos, sino en un espacio plural étnica, social y culturalmente, en el que convergieron marineros, comerciantes, soldados y prófugos de distintas procedencias que desembarcaron en los puertos del continente (Jeha, 2019; Moreno Silva, 2011).

Entre los tatuados, este ejercicio de reproducción del tatuaje estuvo cargado de significaciones positivas relacionadas con el cosmopolitismo, las relaciones personales, la aventura, los roles de género etc. que adquirió un grado de especialización a través de la vida de grupo, donde se poseyó como una institución con sus técnicas, códigos y tradiciones. Los océanos no eran simplemente espacios para la acumulación de capital. Los espacios de confinamiento como barcos en travesías largas, guarniciones militares y prisiones eran los espacios perfectos para proliferar el tatuaje, como se ha dicho, “*pour passer le temps…*”. Allí se favoreció la aparición de lazos de lealtad y solidaridad, de códigos de comunicación, rituales propios, sentimientos de pertenencia y solidaridad, como lo hacían los soldados británicos y los legionarios franceses en las guarniciones de Birmania y África, respectivamente. Esto dio cabida a una tradición marítima radical que hacía del océano una zona de libertad en el que criminales perseguidos y revolucionarios reprimidos se refugiaron y persistieron (Linebaugh, Rediker, 2022). De esto dan cuenta las vinculaciones profesionales del tatuaje en marineros que se marcaban en sus viajes de ultramar con figuras de mujeres, crucifijos, aves, símbolos nacionalistas, anclas, o sus nombres y apodos para ser reconocidos en caso de morir en un naufragio (Jeha, 2019). O como parte de la réplica, como sucedía con los cuerpos de los convictos transportados de Inglaterra a Australia que partían sin marca alguna, y que llegaban a su destino cubiertos con estos, realizados durante el viaje, posiblemente inspirados en los marineros que los transportaban (Anderson, 2004; Alker, Shoemaker, 2022; Moreno Silva, 2011).

Esto recuerda lo narrado por Gabriel García Márquez en *Cien años de soledad* a propósito del regreso de, José Arcadio, el primogénito de los Buendía, tras haberle dado 65 veces la vuelta al mundo con una tripulación de marineros apátridas. De sus viajes no solo quedó el haber naufragado en el mar del Japón, haber acudido al canibalismo para sobrevivir, el vencer a un dragón en el Golfo de Bengala o haber visto en el Caribe la nave fantasma corsaria de Victor Huges:

Llegaba un hombre descomunal. Sus espaldas cuadradas apenas si cabían por las puertas. Tenía una medallita de la Virgen de los Remedios colgada en el cuello de bisonte, los brazos y el pecho completamente bordados de tatuajes crípticos … el cuero curtido por la sal de la intemperie, el pelo corto y rapado como las crines de un mulo, las mandíbulas férreas y la mirada triste. … Hablaba el español cruzado con jerga de marineros. … exhibió sobre el mostrador su masculinidad inverosímil, enteramente tatuada con una maraña azul y roja de letreros en varios idiomas … Las mujeres que se acostaron con él aquella noche en la tienda de Catarino lo llevaron desnudo a la sala de baile para que vieran que no tenía un milímetro del cuerpo sin tatuar, por el frente y por la espalda, y desde el cuello hasta los dedos de los pies (García Márquez, 2023, p.96-98).

De esta manera, podría sugerirse que, si bien los flujos migratorios hacia Colombia fueron menores en comparación con los del sur del continente, el tatuaje fue un elemento introducido al país como una consecuencia a largo plazo de la circulación de viajeros europeos y americanos hacia y dentro de la región a partir del siglo XVI. Los cuales prestaron a los saberes del tatuaje tradicional local las técnicas de tatuado a mano perpetuados en Europa desde la época del Imperio Romano, de los rituales cristianos del siglo IV, así como de las prácticas traídas de la Polinesia por exploradores europeos. Todas ellas consistentes la realización de piquetes en la piel con algún elemento punzante delineando una figura que resultaba en una herida sobre la cual se aplicaba colorante para que el dibujo quedara plasmado de una manera más o menos clara (Guerzoni, 2018; Moreno Silva, 2011).

La evidencia de esta podría extraerse al comparar las Figuras 1, 3 y 4 – y en toda una serie de fotografías de reos de la época (Colección Archivo Casasola, s.f.) – en las que se puede observar que el estilo y los diseños del tatuaje en Colombia en la primera mitad del siglo XX guardan una cercana similitud con los hallados entre criminales, marineros y militares europeos. Los tatuajes de las dos latitudes se caracterizan por una uniformidad en el trazado de figuras con esbozos rápidos y lineales con rellenos simples e imprecisos, con rostros elaborados con carencias creativas y de realismo de las que hablaremos más adelante. Elementos a los que se sumaron las limitaciones materiales que acompañaban a los diletantes tatuadores, como veremos, dotados de herramientas como agujas y tintas diseñadas para otros propósitos, y usados en lugares precarios e insalubres.

## Marcas corporales e imaginarios policiales

Ahora bien, la inmovilidad del tatuaje y su perpetua existencia implica que el mismo es objeto de lecturas diversas que varían su interpretación en la medida que se alejan de su contexto de producción y de las necesidades del individuo en su elaboración. Esto implicó que a partir de finales del siglo XIX la criminología le asignara valores negativos al tatuaje, calificándolos como propios del ocio, la holgazanería, el desempleo y el aburrimiento de los confinamientos por largos periodos de tiempo y sin ocupaciones adecuadas (Gilbert, 2019). Siendo estos los motivos, el tatuaje se convirtió en un emblema visual de la vagancia y del tiempo vital perdido en el vicio, lo cual, para los antropólogos criminales, era un estigma del atavismo identificable con factores culturales y anatómicos (Caplan, 2000b).

La universalidad de esta marca para la identificación criminal fue estudiada con gran interés por la escuela de la antropología criminal italiana, cuyo principal exponente, Cesare Lombroso, era de la opinión que esos adornos, principalmente hallados en miembros de las bajas capas sociales, eran un síntoma externo de degeneración o por lo menos una tendencia a lo primitivo y atávico (Hering, 2019). Como una suerte de exhibicionismo del mal, dependiente de una patología inherente y hereditaria en el individuo, e identificables en determinadas anomalías físicas y psicológicas que dieron origen a su teoría del “delinquente nato”. Una idea debatida por Alexander Lacassagne que, pese a estar de acuerdo con Lombroso en la existencia de factores hereditarios y sociales como determinantes de las conductas criminales, era contrario al concepto de criminal nato (Lombroso, 2006). En su argumento, señaló que estas “cicatrices parlantes” que embellecían a una persona si bien indicaban el carácter, la moralidad y eran un síntoma de vanidad y la profesión elegida en los comienzos de la vida del delincuente, no estaban sujetos a factores hereditarios, sino que tenían su genealogía en el contexto social, en el entorno del individuo, el ambiente y por toda una serie de procesos históricos personales (Lombroso, 2006, p.58-62; Caplan, 2000b; Tatuajes…, 1921).

La irrupción en Colombia de estos nuevos postulados que indagaban sobre las expresiones somáticas del crimen a finales del siglo XIX llevó a varios médicos y abogados a elaborar los primeros cuestionamientos sobre el origen y las causas del tatuaje dentro de los grupos subalternos. Gran parte de estas consideraciones surgieron entre expertos en quienes hizo carrera el ideario positivista italiano. Esa escuela se fue consolidando a través de todo un corpus de autores, y a la vez integrado en tesis de médicos y abogados, así como en litigios y análisis médicos que integraron determinismo criminal en el debate sobre la cuestión criminal (Parada, 2023).

Uno de los primeros en preocuparse por el estudio del tatuaje criminal fue el abogado antioqueño Miguel Martínez. En 1895, consideró que este elemento tan importante para los criminólogos italianos no era entonces un elemento cultural y distintivo del delincuente colombiano, lo cual dificultaba la creación de un biotipo criminal de acuerdo al modelo planteado desde Europa. En lugar de observar tatuajes, “nuestros criminales cuentan con otros distintivos: las cicatrices que son numerosas y en lugares visibles” (Martínez, 1895, p.8). Para Martínez, por el contrario, el tipo criminal colombiano, específicamente el antioqueño, tenía otras características como ojos negros y cabello negro, labios gruesos, rostro prolongado, sin barba, de color de piel moreno, negro o aceitunado y de vigor corporal. Una mirada biológica que asimilaba las relaciones atávicas del tatuaje a dimensiones sociales, morales y raciales colombianas, en la medida que su arquetipo criminal se acercó principalmente al tipo indígena (Martínez, 1895).

La progresiva aparición del tatuaje entre colombianos y extranjeros a principios del siglo XX, y en especial a propósito del caso de los apaches, llevó a los cuerpos de vigilancia a plantearlos como una herramienta a partir de las cuales la policía podría leer la historia de un criminal con solo mirar a su cuerpo. A través del tatuaje, se decía en prensa, se podían relatar historias de traición, venganza, afrentas, amores no olvidados, o cualquier situación capaz de ser interpretable, pues era imposible atar las marcas corporales a reglas fijas. Unas iniciales podrían indicar el nombre de una amante, una llave significaba silencio entre criminales, una cabeza era un símbolo de venganza y de represalia. Frases, puñales, rostros, lagartos, serpientes etc. quienes se decían expertos en el tema apuntaron que llevar todo el cuerpo tatuado era un honor, pues los tatuajes eran condecoraciones expresas entre grupos criminales. Además, fue considerado un atractivo que enloquecía a las mujeres, como lo apuntó el médico cirujano colombiano Víctor Ribón (10 ene. 1919, p.5), pues “su objeto principal es dar al individuo mejor talante o hacerlo más temible”.

Entre tesis que extrapolaban las significaciones, historia y elaboración del tatuaje se encontraron consensos y desacuerdos. La Policía Nacional no era ajena a la presencia del tatuaje en los cuerpos de los pobres miserables que solían capturar sus agentes. Y aunque estuvieran poco acostumbrados a este por la poca frecuencia con la que eran vistos, o porque no eran de la magnitud como la de los apaches franceses, igualmente había un interés manifiesto por aprovechar sus insumos en la identificación. En conferencias dictadas en la escuela de detectives y en publicaciones de la *Revista de la Policía Nacional*, la asociación causal entre criminalidad y tatuaje, aunque variaba entre ponentes, les otorgó a los agentes el rol de reorganizadores de la sociedad a partir del análisis y reconocimiento de categorías y taxonomías criminales verificables en anomalías orgánicas, somáticas, morales e intelectuales del delincuente establecidas por los instructores. Así, entre cabezas puntiagudas, mandíbulas salientes, cavidades oculares desproporcionadas, ojos con estrabismo y narices ganchudas, el tatuaje fue incluido como una manifestación del hombre degenerado y hostil (Paris, 1914; De Toro, 1914).

Esta integración del cuerpo, y en especial del tatuaje, en la agenda punitiva y preventiva del Estado fue grabada en el quehacer policial en los manuales de identificación antropométrica. Así lo propuso Cayetano Méndez (1911), el primer instructor científico y autor del primer ensayo de identificación “Policía Nacional: antropología criminal”. Siguiendo a Lombroso, consideró que los elementos antropológicos eran capaces de revelar tradiciones atávicas, siendo el tatuaje “una primera escritura del salvaje, su primer registro civil” (p.469). El tatuaje era para Méndez (1912, p.70-71) una comprobación del carácter del criminal, carente de sensibilidad dolorífica y táctil, que entre “la raza primitiva sudamericana”, canibalesca, supersticiosa y falta de sentido moral, era un martirio ejecutado para satisfacer la vanidad.

Menos extensos teóricamente, pero igualmente preocupados por la necesidad de registro del tatuaje, fueron las obras *Conocimientos generales sobre antropología, antropometría y dactiloscopia*, de José Gregorio Puentes (1912); *Identidad judicial: importancia de los gabinetes antropométricos*, de Aníbal Cuartas (1917), que comparaba la práctica antropométrica en Medellín con la de la policía de Barcelona tras un viaje de estudio a España; y la *Identificación personal: dactiloscopia*, de Rafael Colmenares del Castillo (1949), el tatuaje tenía vital importancia en la identificación.

Hacia finales de la década de 1910, el capitán Osuna, instructor y dactiloscopista más familiarizado con el tatuaje que los demás miembros de la policía colombiana, publicó su propio manual de identificación antes de terminar su misión en Colombia en 1919. Y ya que había dirigido el caso de los apaches y había permanecido “cerca de cuatro horas … a brazo partido con ‘el fiambre’”, es decir, con el cadáver de Georges Petit, dedicó unas páginas al tatuaje, pues ya lo había visto en apaches, bandoleros andaluces, miembros de la Legión Extranjera Francesa y en las galerías criminales de las policías del continente europeo (Basabe, 2020). Sus comentarios sobre el caso brindan valiosos detalles frente a estas marcas tanto por expresión artística reconocida como producto de una tradición cultural visual de largo aliento, así como por ser una marca que ofrecía insumos de interpretación en los procesos de identificación.

Señaló, entre otros, que este era un producto original de la Polinesia, adoptado por marineros y exploradores durante sus recorridos comerciales hacia oriente. Que era generalmente hecho con punzadas de agujas afiladas que dejaban una herida a la cual se aplicaba generalmente carbón, pólvora y hollín en lugar de la actual tinta, y que tras el procedimiento era lavado con orina (Osuna, 1917). Así lo detalló:

Suelen encontrarse en gentes de baja condición, degenerados, viciosos, marineros, soldados, presidiarios, prisioneros y apaches, siendo estos últimos los más aficionados a ellos … En cambio, las mujeres son muy refractarias a los tatuajes, especialmente las que comercian con su hermosura. En todo caso, y calificando favorablemente, el tatuaje es un signo de degeneración, vicio, exaltación instintos criminales o capricho estúpido. No se comprende como las personas que un día u otro tendrán que habérselas con la justicia y con la policía, se ponen caprichosamente en su cuerpo ciertas marcas inconfundibles que facilitan extraordinariamente su persecución e identificación … por su variedad y su fijeza, toda vez que son muy difíciles de quitar. … Del tatuaje puede deducirse a veces la profesión de individuo, sus pasiones, sus instintos y sus deseos: en los marineros suelen encontrarse un ancla o una virgen; en los presidiarios una cadena, un nombre o una fecha; en los apaches, retratos de sus mujeres, armas, nombres y atributos de sus vituperables ocupaciones; en los peregrinos imágenes de santos; en los estetas, símbolos nefandos y leyendas repulsivas; en los que son presa de idea de venganza el nombre o la figura de su futura víctima, o un recordatorio; en los soldados, armas de guerra, nombres de generales y batallas … y en los ricos o nobles, que se tatúan por pura extravagancia, escudos heráldicos, escenas de cacerías o deportes y árboles genealógicos … Como tatuados célebres pueden citarse el difunto rey de Inglaterra, Eduardo VII, que durante muchos años fue el árbitro de la moda; el ex zar de todas las Rusias, Nicolas II; el ex sultán de Turquía, Abdul Hamid; el mariscal Lefevre; Bernadotte, y el pretendiente a la Corona de España don Jaime de Borbón, cuyos preciosos tatuajes en la espalda, pecho y brazos he visto yo (Osuna, 1929, p.297-304).

Este análisis, de quien entonces era el máximo referente científico de la Policía Nacional, establece unos lineamientos entre lo permisible y lo peligroso dentro del tatuaje, definidos por vectores de clase, estética y moral, restringiéndolos a límites racionalmente aceptados. Con estas diferenciaciones entre estigma y estilo, o a lo sumo como “frivolidad mundana”, se identifica una separación discursiva entre clases burguesas, inmunes a la degeneración y los vicios, frente a las clases menos favorecidas, proclives al salvajismo (Jeha, 2019). El tatuaje no era pues solamente una marca física, sino también una tecnología de vigilancia para determinar rasgos de pertenencia y exclusión. Era, siguiendo a Osuna, un elemento capaz de establecer tendencias de moda que en la práctica generaron un paradigma honorífico de damas y caballeros tatuados en las personalidades de reyes, reinas y príncipes, como el rey Carlos XIV de Suecia y Noruega, que llevaba tatuado en el brazo el lema “*Mort aux rois*” (Muerte a los reyes) (Rocha, 2022, p.80; Williams, 2022).

Con esto, es posible reconocer que la mirada científica de los cuerpos de vigilancia colombianos en proceso de profesionalización aprendió de Osuna a diferenciar entre lo peligroso, en los tatuajes de unos extraños desheredados, y lo bello, en los tatuajes de grandes dignidades como don Jaime de Borbón, cuyos tatuajes no fueron objeto de crítica o análisis criminológico alguno durante su visita al país tan solo unos meses después del caso de los apaches (La visita…, 19 jun. 1920).

## Dos versiones sobre el tatuaje en Colombia

Como hemos venido señalando y como se ahondará en este apartado, al igual que Lombroso (2006) en Italia, Rafael Salillas (1908) en España, Francisco Martínez Baca (1899) en México o Walther Schönfeld (citado en Ebergard, 2015) en Alemania, muchos médicos, policías y abogados en Colombia aprovecharon calabozos y celdas en presidios y estaciones de policía como laboratorios de experimentación y análisis del tatuaje, en donde realizaron sus propias lecturas y registros de las marcas en los individuos encerrados.

El cuerpo marcado tenía una capacidad enunciativa en el ámbito punitivo y médico-legal, donde se ejecutaban ejercicios de análisis sobre el individuo tatuado, tanto desde aspectos biográficos, de vital importancia para la identificación, como clínicos, en la medida en que a partir de ellos era posible emitir diagnósticos de enfermedad y desviación y su impacto dentro de nociones de raza, criminalidad, masculinidad y ciudadanía. Allí, como veremos, la exterioridad era traducida, seleccionada y teorizada de acuerdo a las proyecciones del tatuaje, usando como regla somática y gramatical el ideal del cuerpo normal, aquel que sin la marca del tatuaje no emitía discursos de agencia y podía pasar ignorado por la historia (Putzi, 2006; Cardona, 2012).

**Figura 2:**
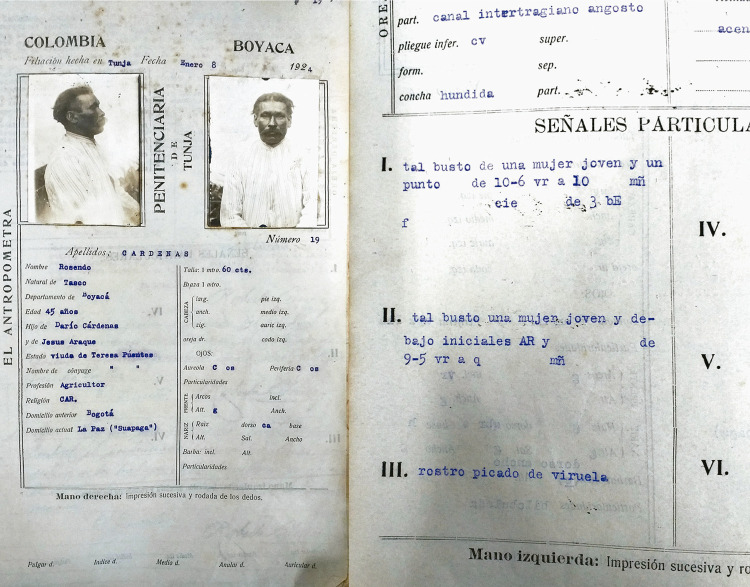
: Fichas antropométricas con descripciones de tatuajes del Panóptico de Tunja (Penitenciaría de Tunja, 1924)

Estos expertos dejaron importantes registros de las figuras, estilos y técnicas en filiaciones y fichas antropométricas durante la primera mitad del siglo XX, las cuales ofrecen una pequeña pero considerable muestra que da cuenta de la perenne proliferación del tatuaje, principalmente, entre individuos, sujetos marginalizados y sometidos a la vigilancia policial y penitenciaría desde la década de 1910. En estas se han hallado a menores de edad con tatuajes de cruces, corazones y espadas hechos con tinta china, registrados por la policía de Medellín en 1917 (Cuartas, 1917); agricultores boyacenses, albañiles tolimenses, alfareros vallunos y jornaleros venezolanos marcados con iniciales, cruces de malta, palomas, bustos de mujeres y cristos de hasta veinte centímetros, registradas bajo la abreviatura “tal.” en 1924, en el Panóptico de Boyacá tras su llegada del Panóptico de Ibagué (Figura 2) (Penitenciaría de Tunja, 1924); y presos del Panóptico de Manizales con tatuajes de culebras y mujeres desnudas en el mismo periodo (Penitenciaría de Manizales, 1924).

En la década de 1930, en Casa de Menores y Escuela de Trabajo de Bucaramanga, el tatuaje era realizado desde muy temprana edad en menores encerrados que se tatuaban los unos a los otros. Para el director y los maestros de ese centro de nada servía el trabajo manual y agrícola, ni las conferencias sobre moralidad impulsadas, las anormalidades del comportamiento de los menores quedaban de manifiesto en la práctica con el tatuaje, al cual reconocieron como una marca de “vagancia”, “perniciosidad” y “peligrosidad” (Ortiz, 2012, p.208). De la misma manera, en la cárcel de mayores de esa ciudad, el tatuaje era estudiado detalladamente: primero, para escudriñar en la vida del delincuente y plantear hipótesis sobre las causas del delito en el mismo; y segundo, para capitalizar la corporalidad y atar a los individuos no tatuados al aparato productivo, seleccionando solamente los cuerpos no marcados para las labores de vigilancia por la garantía de honorabilidad que esto significaba (Cardozo, 2006).

Estos fueron apenas unos registros iniciales cifrados en datos que representaban la información visual en nomenclaturas exclusivas por y para el personal de los gabinetes antropométricos durante el proceso de identificación y registro corpóreo de reos. Ahora bien, aunque a partir de la década de 1910 la vulgarización de la antropometría y su capacidad de hallar evidencias permite identificar las temporalidades del tatuaje, las fichas producidas en sus gabinetes tienen la desventaja de no incluir fotografías o recreaciones de los tatuajes en croquis ilustrativos como lo hacían sus pares en el mundo (Cabellud, 1908). Lo cual limita el deseo por identificar en ellos cambios, continuidades y maleabilidades de la manera que se puede hacer con el tatuaje posterior a la década de 1930, cuando se realizan los primeros estudios sobre el mismo.

A partir de este periodo las trasgresiones evidenciadas por los observadores del cuerpo tatuado fueron clasificadas para crear taxonomías con las cuales distribuir racionalmente a los marcados en categorías de criminalidad y enfermedad. Aunque fueron posiblemente muchas las publicaciones hechas en torno a este tema, esta investigación ha hallado como fuente documental primero *El tatuaje de la piel y sus distintos aspectos en la criminología*, escrito por José María Garavito Baraya (1966), jefe del Laboratorio Forense del Instituto Colombiano de Medicina Legal; y segundo, en *El tatuaje en Antioquia*, publicado por Alfredo Correa Henao (1965).

Garavito Baraya era un médico especialista en investigación criminalística y forense, cuya trayectoria profesional fue dedicada al peritaje judicial en la averiguación de hechos criminales a través de estudios toxicológicos, balísticos, radiográficos, valoraciones de lesiones personales y necropsias, donde se encontró varias veces con el tatuaje. La piel tatuada, su histología, su elaboración y su relación con determinadas manifestaciones biológicas fue estudiada por Garavito en los cuerpos de individuos asesinados violentamente cuyos cadáveres no reclamados reposaban en el Instituto de Medicina Legal y al Laboratorio de Criminalística de la Universidad Nacional. Procurando consérvalos para sus clases de medicina forense y para estudios posteriores, Garavito extrajo de los cadáveres algunos trozos de piel tatuada con un procedimiento de curtido para preservar la piel tatuada recién arrancada del cuerpo, cuidando gran parte de la naturalidad de la piel y colores del tatuaje, y que fue montando en tablas pulidas y protegidas con plástico. Una práctica al parecer común entre algunos médicos, ya que, por ejemplo, Mario Robledo Villegas contaba con su propia colección para el estudio de lesiones cutáneas (Garavito Baraya, 1966; Correa Henao, 1965).

**Figura 3:**
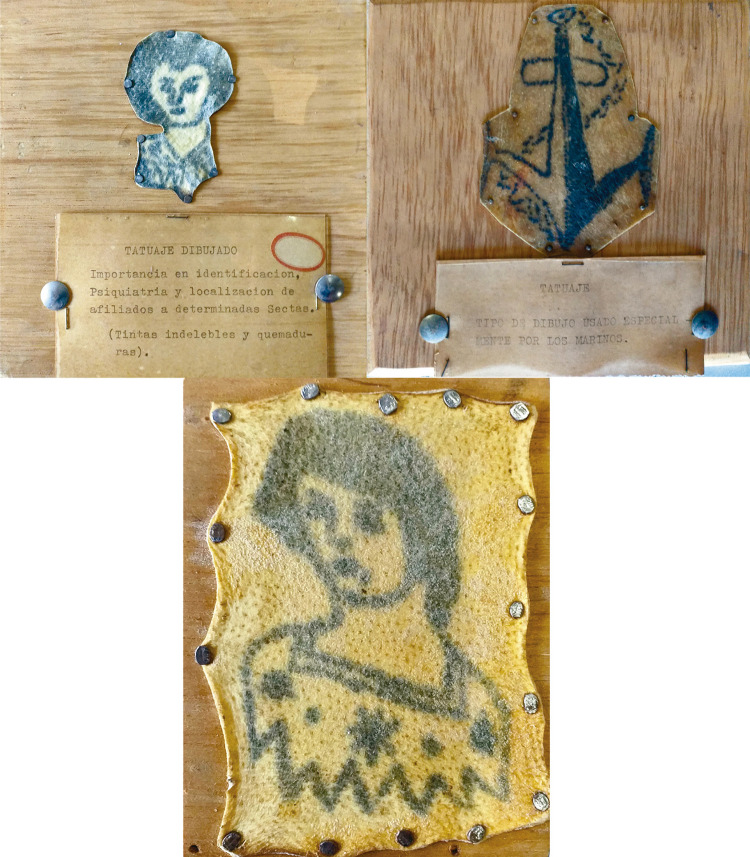
: Colección de tatuajes de José María Garavito Baraya tomados de los cadáveres de dos extranjeros (Colección Museológica…, s.f.)

Esta impresionante colección de cerca de 12 tatuajes criminales llegó a conformar toda una red de museos criminológicos de tatuajes entre los que se puede contar la del médico patólogo japonés Masaichi Fukushi con más de dos mil piezas; la del español Rafael Salillas expuesta en la Exposición Penitenciaria de San Petersburgo (1890); el Museo Anatomopatológico de Ángel Ferrer, con cerca de seis mil tatuajes; la colección de tatuajes de Instituto Nacional de Medicina Legal e Ciências Forenses de Portugal, entre otras. Las cuales encuentran como puntos en común el interés médico criminológico por contemplar en el tatuaje la proliferación de enfermedades, como los reflejos y efectos de las intoxicaciones crónicas, los efectos de la sífilis o la tuberculosis. Pues se les consideraba, además de una temeridad, un síntoma de algún tipo de enfermedad, de predisposición al crimen o de atavismo.

Los cadáveres recibidos, los tatuajes recortados y su experiencia forense lo guiaron a conclusiones más o menos concretas sobre el tatuaje, aunque no fueran estas sino una colección de epidermis ilustradas que documentaban frágilmente la vida del tatuado. Garavito Baraya era de la idea de que esto no era algo común entre los colombianos. Aunque desde finales de la década de 1910 eran muchas las fichas policiales e informes médicos que registraban tatuajes incluso entre campesinos y jornaleros de poblaciones lejanas, como Saboyá en Boyacá o Piedras en Tolima (Penitenciaría de Tunja, 1924), su experiencia le indicó que el tatuaje era algo visible específicamente en extranjeros, un elemento que adquirió mayor presencia con el aumento de las migraciones producidas por los conflictos bélicos internacionales (Jeha, 2019). Muchos de estos expuestos en fotografías de sus rostros y sus tatuajes en las galerías de “Extranjeros indeseables” de la *Revista de la Policía Nacional* publicadas desde la década de 1920 y en las “Galerías de ratas” o “de cacos” que había en algunas estaciones de policía en el país (Una galería…, 20 sep. 1929, p.1). También fue un elemento introducido en la literatura nacional de principios del siglo XX, como recordó en 1924 José Eustasio Rivera (2015, p.153) en *La vorágine* con la figura de El Pipa, un indígena “vanidoso de sus tatuajes y cicatrices, prefería el guayuco a la vestimenta” que había sido encarcelado en múltiples ocasiones, donde pudo adquirir tales marcas.

Pese a la evidencia que señalaba que el tatuaje era practicado entre los campesinos tolimenses y los caucheros de lo más profundo del Amazonas, apuntó Garavito Baraya (1966), el tatuaje solo era visto en una reducida cantidad de colombianos que habían vivido fuera del país o que habían sido miembros de legiones extranjeras. Desde su trayectoria profesional fijó un nexo entre el factor delincuencial y el tatuaje por ser uno de los elementos característicos que había hallado en sujetos, principalmente hombres, asesinados en riñas callejeras, así como en suicidas, más que en muertos por otras causas. De ahí que le fuera posible reflexionar en torno a la sociología del tatuaje y al carácter de los tatuados, clasificándolos por el tipo de tatuaje así:

a) las obras de arte en varios colores son comunes en los ingenuos, débiles de carácter y sin personalidad … d) los dibujos pornográficos, en los pervertidos sexuales; e) con aspectos masculinos, en los pederastas; d) con recargados adornos, instrumentos musicales y otros semejantes, en las mujeres de vida alegre. El hecho de tatuarse o permitirlo es síntoma de un estado sicopático, como es el caso de los asténicos, excitables, paranoicos, hipertímicos e hipotímicos, sicoasténicos, histéricos y pervertidos sexuales. … También podemos dividir a los tatuados, aunque exageradamente, en anormales y costumbristas o disciplinados, correspondiendo este último grupo a los marinos y soldados de algunas otras armas en quienes es tradicional su uso (Garavito Baraya, 1966, p.46-48).

A partir de esto catalogó a los delincuentes tatuados entre anormales, costumbristas y disciplinados, sin especificar las diferencias entre uno y otro. Con esto ordenó a los individuos de acuerdo a las patologías que iba encontrando y a las intoxicaciones crónicas que identificaba. Así pues, aunque dentro de su teorización concluía que el tatuaje no era necesariamente indicativo de la personalidad del tatuado, apuntó que de allí podían extraerse reflejos de sus distintivos psicológicos. Por ejemplo, los tatuajes en las zonas genitales los asoció a los pederastas, y las estrellas, medallas o condecoraciones los indicó como símbolos de diferenciación y estatus dentro de las mafias (Garavito Baraya, 1966).

Un año antes del estudio de Garavito, el médico patólogo Alfredo Correa Henao reunió, en un análisis más detallado, sus observaciones sobre los tatuajes, los cuales fue observando y coleccionando, no ya en trozos de piel sino en dibujos calcados por él mismo en los servicios médicos municipales, en la Cárcel de Varones de Medellín y en el Instituto de Anatomía Patológica de la Facultad de Medicina de la Universidad de Antioquia. En *El tatuaje en Antioquia*, Correa Henao (1965) estudió y clasificó más de cien tatuajes tomados en pechos, brazos, escápulas, muslos, penes y rostros desde la década de 1930. Con esto verificó la evolución de determinados tipos predominantes de tatuajes que permiten comprender, entre otros, el oficio del tatuador, las fuentes de los diseños tatuados, las relaciones particulares entre los sujetos, tradiciones, técnicas etc.

Correa Henao (1965) señaló que el tatuaje contemporáneo estaba marcado por sendas diferencias con el tatuaje étnico, típico entre los “pueblos primitivos”. El segundo, era para él una muestra de la cultura, tradiciones, honores y proezas de los pueblos indígenas, un elemento con significaciones rituales impresas en el carácter y un talismán protector. Mientras que el primero era propio de miembros de las más bajas categorías sociales e intelectuales, de aventureros y trashumantes “muy frecuentemente con taras síquicas y con inclinaciones perversas” (p.461). Destacando entre ellos braceros portuarios, limpiabotas, marineros, soldados, pescadores, o más específicamente hampones, presidiarios y prostitutas.

Para Correa Henao (1965, p.462), el tatuaje era propio de las clases sociales sedimentarias mal reputadas, es decir, “individuos jóvenes, inexpertos; sujetos tarados síquicamente, con perversas inclinaciones; aventureros errabundos etc.” sobre los que obraban factores morbosos que los impulsaban al tatuaje. Entre estos, el homosexualismo, las pasiones exageradas, los sentimientos familiares, amorosos y, dada la alta religiosidad de los antioqueños, las pasiones religiosas. Las cuales, señala Correa Henao, llevadas sicopáticamente, podían hacer creer al individuo que el tatuaje podía patentizar de forma altamente expresiva los diversos sentimientos como si estuvieran grabados “en lo profundo del corazón” (p.462).

Ahora bien, lo que diferencia a la investigación de Correa Henao de lo elaborado por Garavito Baraya y de los análisis criminológicos ya mencionados, es que su investigación no solo abordó al tatuaje como un cifrado de anomalía, sino que indagó frente a los contextos históricos y visuales en los que se desarrolló el tatuaje, sobre sus valores estéticos, las figuras del tatuador y del tatuado, y los patrones visuales que influenciaron diseños y tendencias, acercándose al mismo como una muestra de arte popular y como un ornamento.

Así, el tatuador antioqueño de la época, a diferencia del tatuador tribal respetado en su comunidad por ser el agente transmisor de tradiciones culturales entre generaciones, era en palabras de Correa Henao un “culebrero”*.* Esto es, un vendedor callejero comúnmente visto por pueblos y ciudades trepado en carros o caminando, a veces cubierto de plumas para vender remedios y brebajes para la mordedura de serpiente como contó Luis Zea Uribe (28 mar. 1919), pero casi siempre visto semidesnudo exhibiendo su cuerpo tatuado como forma de reclamar su arte. Se paseaba por plazas, mercados, estaciones, ferias y barrios bajos mostrando sus colecciones de tatuajes dibujados en papeles con diversos diseños y posibles significaciones según las necesidades y emociones del cliente. Su labor, cuando no en presidios, calabozos o cantinas, era ejecutada al aire libre, ante la atónita mirada de los curiosos, por lo que las infecciones de tétanos, sífilis, hepatitis y gangrena eran comunes (Correa Henao, 1965).

Como se trataba de una técnica barata, cuando no pactada como parte de un intercambio de bienes, el precio se tasaba de acuerdo con temas, diseños, tamaños, y en pocos casos el uso de colores, que eran por lo demás bastantes precarios (Moreno Silva, 2011). La técnica, como hemos dicho, era la tradicional marcación mediante punciones con agujas untadas con colorantes aprendida de las técnicas nativas y de los viajeros extranjeros. El proceso era sencillo, las imágenes eran calcadas en un papel y luego aplicadas sobre la piel previamente humedecida para garantizar que la tinta marcara las figuras dibujadas por el tatuador. El resultado dependía de la expresión y sensibilidad del artista porque, en el caso de las siglas y números, estas no eran calcadas de un papel sino dibujadas a pulso directamente sobre la piel. Marcado el boceto, dos o tres agujas de coser comunes, atadas con un hilo, eran humedecidas en tinta china generalmente, aunque también podía ser pólvora u hollín de caucho quemado disuelto en agua. Con este material, se iniciaba la punción sobre la línea del dibujo, atravesando la epidermis y depositando el colorante en la dermis (Correa Henao, 1965).

El individuo tatuado colombiano durante este periodo tenía una característica particular, de acuerdo a Correa Henao. A diferencia del tatuado japonés, cuyo tronco y extremidades podían estar cubiertas por la más finísima y experta obra de arte, el estilo del tatuaje colombiano era más bien precario, con máximo dos tatuajes. Para Correa Henao, las personas tatuadas en Colombia eran individuos “simples” de pueblo, arquetipo completamente opuesto al extranjero o al colombiano cosmopolita que describió Garavito Baraya. Y si bien era más común ver el tatuaje en hombres que en mujeres, quizás por las convenciones sociales de la época o por la negativa de muchas a mostrar su cuerpo, había un reducido número de mujeres tatuadas, entre ellas prostitutas que se tatuaban lunares en el labio superior o la mejilla como marca de sensualidad, y otras pocas presas comúnmente tatuadas con las iniciales de sus amados (Correa Henao, 1965; Rocha, 2022).

En general, para Correa Henao (1965), el tatuaje colombiano no era nada espectacular. Aunque su elaboración requería el aprendizaje de un saber, para él eran, en su mayoría, dibujos simples hechos por un inexperto, y en pocos casos temas adecuadamente reproducidos. Así, el individuo tatuado colombiano se marcaba tanto con complejos simbolismos reales o imaginarios “que solo una mente perturbada querría tener de por vida”, como por figuras “pueriles e ingenuas” sin significación alguna (p.468).

A partir de sus experiencias cercanas al tatuaje, Alfredo Correa elaboró una serie de teorías y categorías de análisis en torno a su realización bajo una lectura iconográfica de los signos corporales, ponderada con categorías geográficas, sexuales, morales y religiosas, influenciada por los debates criminológicos europeos. Así, creó un índice de tipos de tatuajes separados por delgadas líneas que permitían al tatuado permanecer en más de una a la vez. Y al igual que Garavito, dejó en claro que no siempre era posible lograr asociar un tatuaje a un estado psíquico, a algún reflejo de la interioridad del individuo o a las intenciones propias del tatuado, significando que el tatuaje por sí solo no era suficiente para categorizar a un individuo, sino que a este debían sumarse el análisis de otros factores externos e internos.

Y aunque el individuo se tatuara simplemente por plasmar en su piel valores religiosos, sentimentales, derivados de su labor o, como decían los apaches, “por pasar el tiempo…”, médicos, abogados y policías lo abstrajeron de su carga estética para revelar en ellos caracteres delincuenciales y muestras de criminalidad patologizada. Como indicó hacia mediados del siglo XX el criminalista colombiano Luis Carlos Pérez reproduciendo el parentesco lombrosiano que relacionaba tatuaje, salvajismo y crimen:

El tatuaje es una comprobación frecuente del tipo criminal. El mayor número de tatuados lo dan los reincidentes y los criminales natos, sean ladrones o asesinos; el menor, los falsarios y estafadores. Las causas del tatuaje son: la religión, la imitación, la venganza, el ocio y la vanidad, pero, sobre todo, el atavismo, como reproducción de una costumbre difundida entre los salvajes, con quienes el delincuente tiene tanta afinidad, por la violencia de las pasiones, lo torpe de la sensibilidad, la vanidad pueril y el ocio prolongado; y también por el atavismo histórico, como sustitución de una escritura con símbolos a la escritura común alfabética (Pérez, 1947, p.177).

**Figura 4:**
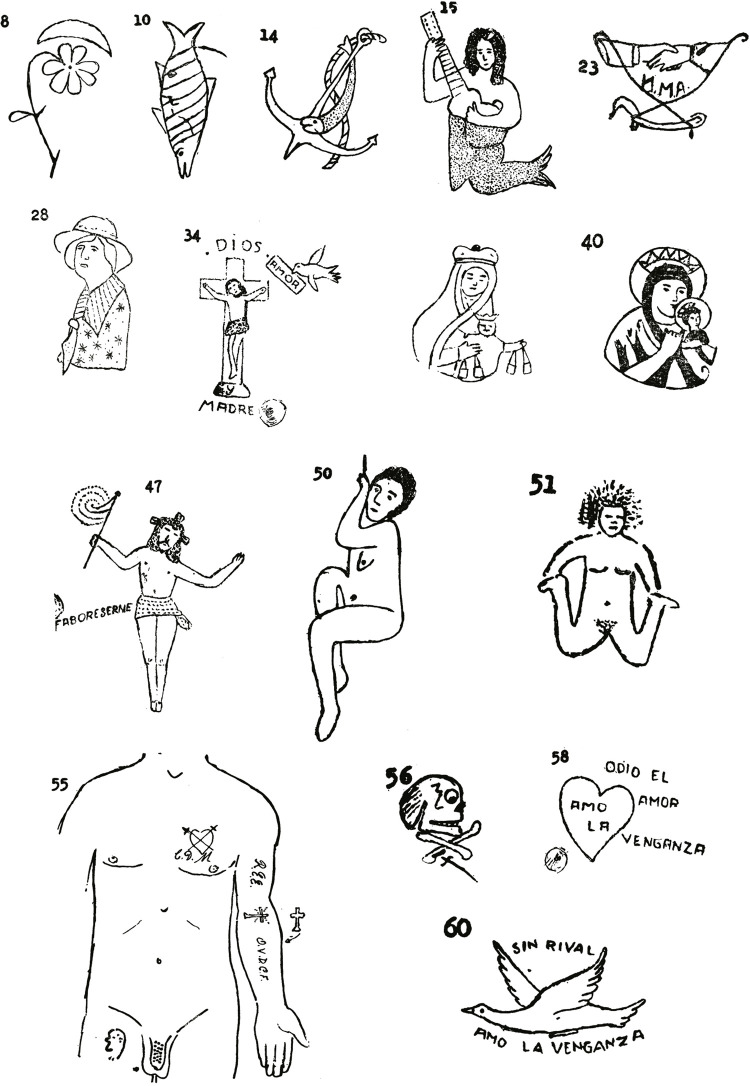
: Colección de tatuajes de Alfredo Correa Henao (1965)

De esta manera, Correa Henao (1965) clasificó el tatuaje entre ornamental, amoroso, religioso, erótico y violento. Siendo el primero aquellas figuras (flores, animales, figuras populares etc.) (Figura 4, números 8 y 10) sin significaciones aparentes, aunque cargados con expresiones de misticismo (cruces) o romanticismo (medias lunas). Con este, Correa Henao dio cuenta de la apropiación de este saber en Colombia a través de prácticas extranjeras, ya que era el tipo más sencillo de grabarse en la memoria, como sucedía en la zona costera del país, donde era común ver tatuajes de sirenas y anclas no entre marineros y pescadores como parte de su profesión, sino como resultado de una imitación, influenciada por lo visto en marineros tatuados provenientes de otros países (Figura 4, números 14 y 15).

El tatuaje religioso fue considerado por Correa Henao el tipo de tatuaje más popular en Colombia, cuya proliferación se debía al deseo de los individuos por poseer la representación visual de los eventos y personajes de la tradición católica, cuya capacidad de conmoción los llevaba a la realización de transformaciones físicas, “produciendo una rica y multidimensional fenomenología de la experiencia religiosa” (Moreno Silva, 2011, p.188). Entre estos destacaban iniciales (J.M. y J. [Jesús, María y José] y JHS [Jesús Hombre Salvador]), figuras de la Virgen del Carmen y del Perpetuo Socorro, signos de la Pasión, sudarios, plegarias y señores caídos de Girardota y Monserrate (Figura 4, números 34, 40 y 47). Los cuales, para Correa Henao (1965, p.477), eran propios de “gentes simples, ignorantes, crédulas o sicópatas que piensan que esta manifestación religiosa, grabada físicamente, impetraría con más eficacia al auxilio y protección sobrenaturales”.

Junto a estos estaban los tatuajes amorosos y eróticos (Figura 4, números 23, 28, 50 y 51), surgidos de las pasiones candorosas, de los romances inolvidables representados con iniciales, corazones y rostros de mujeres pocos parecidos a la realidad por la incapacidad del autor por retratar la realidad, con los que se representaban sentimientos de desengaño y frustración (Correa Henao, 1965). En los motivos desnudos y sin detalles, principalmente hechos en cárceles, por ser la soledad de las celdas donde más se exacerbaban las pasiones sexuales, el médico observador pudo evidenciar tendencias o móviles sexuales que cumplían eficazmente la estimulación de la primitiva imaginación del tatuado. En el erótico se manifestaban patológicamente los complejos psíquicos del penado, por lo que el reconocimiento de uno durante una observación médica era significativo en el descubrimiento de desviaciones patológicas sexuales. Por ejemplo, para Correa Hernao (1965), un tatuaje en el pene o en la zona de la pelvis era un elemento frecuente en invertidos sexuales (Figura 4, número 55). Idea soportada por otros médicos legistas como Julio Ortiz Velázquez y Agustín Piedrahita (31 ago. 1931), que lo consideraron una señal evidente del carácter masoquista del invertido sexual constitucional y hereditario, único individuo capaz de ser excitable frente al propio dolor.

Estas fueron las primeras categorías establecidas por Correa Henao durante la fase inicial de sus estudios, que comprendieron desde principios de la década de 1930 hasta finales de la de 1940. Ahora bien, algunos años después, el periodo de la violencia y sus funestas consecuencias materiales y emocionales para la sociedad colombiana hicieron que sus pesquisas identificaran un incremento en los tatuajes con simbolismos violentos. Un tipo no muy recurrente que en su momento no llamó su atención, pero que tras este periodo empezó a hacerse más presente y que, para Correa Henao, era un evidente reflejo del efecto que tuvieron en la piel de los colombianos, las violentas agresiones políticas, la zozobra, el sadismo y la venganza característicos de finales de la década de 1940 hasta la de 1960. El componente del odio se agregó a los diseños de los tatuajes, y su continuo hallazgo llegó a una cifra tal que debió reunirlos en una categoría que en grupo predominaban frente a las ya mencionadas.

El tatuaje violento (Figura 4, números 56, 58 y 60), como lo denominó Alfredo Correa Henao (1965), era frecuente entre individuos varones presos por crímenes sangrientos, como lo evidenció en sus observaciones en reos de la cárcel de Medellín. Donde, por lo demás, los tatuajes eran hechos por un mismo autor, como lo evidenciaban la caligrafía, tipo de letra y trazados que predominaron allí. Los ejemplos presentados, sin referencias políticas identificables, hablaban por sí solos por su rudeza y capacidad de demostración en las que se hacían presentes las ideas de venganza, muerte y asesinato, que para el patólogo representaban perturbaciones psíquicas por las coyunturas sociopolíticas.

Estos tatuajes surgidos como consecuencia social de las retaliaciones partidistas se vinieron a sumar otra serie de tatuajes violentos identificados por Correa Henao desde la década de 1930 en los que hacían gala los motivos bélicos y religiosos. Un ejemplo de este tatuaje es recogido por Correa Henao de la novela *Risaralda* (1935) de Bernardo Arias Trujillo, donde se aborda la idea del bandolero, arquetipo del crimen, el honor, la masculinidad y portador de tatuajes alusivos a su vida criminal, así:

Pedro Antonio Escobar, su segundo y favorito, quitó de la garganta del patrón el escapulario donado a la viejecita ausente y se lo guardó junto con las monedas. Al desprenderle la prenda sacra, apareció el ancho pecho peludo condecorado de tatuajes. Tenía una Virgen del Carmen, patrona de los cuatreros, bien diseñada sobre la mamila izquierda, coincidiendo la cabecita del Niño Dios con el lunar carnoso de la tetilla. La estatua era un prodigio de filigrana y de miniatura. En el duro bíceps derecho estaba dibujado un corazón terriblemente herido por un puñal siciliano cuya punta chorreaba sangre de celos y de venganza. Al pie del corazón, en letras claras, se leía esta frase veraz: ‘de la cárcel se sale un día; del cementerio, nunca’ (Arias, 1960, p.199).

Calaveras con huesos cruzados, puñales con iniciales, aves nada parecidas a la paloma de la paz, calificativos “sin rival” y “amo la venganza” lo llevaron a concluir que el tatuaje era un hecho histórico marcado por elementos atávicos, emocionales, geográficos y sociales, venido desde tiempos antiguos, muy presente en tribus “salvajes” y en la sociedad colombiana de mitad de siglo. Además, como algo característico de individuos ignorantes y de baja extracción social, y que no tenía nada especial en su ejecución o sus motivos, a excepción del tatuaje violento, el cual tenía unas características propias moldeadas por las coyunturas que vivía el país. En resumen, para Correa Henao (1965), el tatuaje era causante de malas reputaciones y estigmatizaciones que asociaban al tatuado con ignorantes y perturbados mentales.

## Consideraciones finales

El caso de Georges Petit ha invitado a establecer una serie de cuestionamientos en torno al tatuaje que, por una parte, abordan la racionalización de estigmas que tamizaron al tatuaje como un elemento de diferenciación identificando el uso de determinados enfoques criminológicos, higiénicos y raciales; por otra parte, a partir de ellos surgieron otros interrogantes en torno a las temporalidades del tatuaje en Colombia, a sus formas y razones de expresión y sus espacios de elaboración, abriendo un debate en torno a la circulación de este saber y de toda su cultura material.

Al igual que en muchas partes del mundo, como se ha dicho, la historia del tatuaje en Colombia sería hoy día menos conocida y accesible de no ser por las extrapolaciones teóricas y el interés que en él mostraron policías, periodistas, médicos y abogados desde principios del siglo XX. Sus asiduas preocupaciones se han reunido en un limitado pero provechoso archivo compuesto, primero, por formulaciones y debates escritos que giraron en torno a los síntomas físicos de la criminalidad, la enfermedad y las desviaciones. Y segundo, con una impresionante colección de imágenes de tatuajes y de recortes de piel tatuada presentados no como simples objetos de archivo, como libros o papeles, sino como rastros de vidas de muchas personas, de memorias de amores y familiares, de experiencias, de creencias religiosas y de estados de ánimo inscritos en la piel. Con estas dos fuentes ha sido posible hacer un sucinto acercamiento a la historia del tatuaje, abriendo además un debate en torno a las formas de interpretación y lectura del mismo desde la perspectiva del académico interesado en los caracteres somáticos del criminal, y con las breves experiencias de los tatuados.

Con esto, se ha observado cómo, desde la primera mitad del siglo XX y hasta la actualidad, la marcación corporal tuvo un rol esencial en la configuración de imaginarios en torno al crimen y de dispositivos para su identificación y prevención. Por lo cual, dentro del poder correctivo del Estado, en gabinetes antropométricos, presidios y hospitales, fueron implementadas unas claves de lectura del mismo, dada su capacidad enunciativa, para facilitar los mecanismos de gobierno de los cuerpos, de filtración de la población, de capitalización de la corporalidad y de definición del modo de ser adecuado. A partir de estas consideraciones que vieron en el tatuaje una práctica atávica, se ha podido evidenciar la práctica del tatuaje durante este periodo como una expresión de las clases sociales subalternas, las cuales, a través del aprendizaje e imitación de la técnica del tatuado, facilitaron el nacimiento de una cultura visual dentro de comunidades y espacios marginalizados.

## Data Availability

No están en repositorio.
